# Successful Treatment of Pediatric Acute Myeloid Leukemia Presenting with Hyperbilirubinemia Secondary to Myeloid Sarcoma: A Case Report

**DOI:** 10.3390/children9111699

**Published:** 2022-11-05

**Authors:** Hwazen A. Shash, Ashraf M. Khairy

**Affiliations:** 1 College of Medicine, Imam Abdulrahman Bin Faisal University, Dammam 31441, Saudi Arabia; 2 Department of Pediatrics, King Fahad Hospital of the University, Al-Khobar 31952, Saudi Arabia; 3 Department of Pediatric Hematology/Oncology/Bone Marrow Transplantation, King Fahad Specialist Hospital, Dammam 31444, Saudi Arabia

**Keywords:** myeloid sarcoma, jaundice, childhood leukemia, chloroma, acute myeloid leukemia

## Abstract

Myeloid sarcoma (MS), a tumor consisting of myeloid blasts with or without maturation, occurs at anatomical sites other than the bone marrow. MS of the gastrointestinal tract presenting with jaundice in children is rare. We report the case of a 4-year-old boy with a 6-week history of symptoms of obstructive jaundice due to a peripancreatic mass compressing the common bile duct. Six weeks later, blasts were found in a peripheral smear prior to surgical biopsy; bone marrow evaluation and flow cytometry results led to a diagnosis of acute myeloid leukemia (AML) with MS. No further invasive testing or temporary drainage was performed. He was started on induction therapy with full therapeutic doses of cytarabine, dose reductions of etoposide, and escalating doses of daunorubicin. His liver enzymes normalized, and he completed subsequent cycles of chemotherapy with full doses. The abdominal ultrasound showed resolution of the mass after the second cycle of chemotherapy. He is currently in remission three years after completing therapy. AML-directed chemotherapy in patients with obstructive jaundice secondary to MS may be beneficial without requiring invasive testing or temporary drainage procedures. Daily follow-up is crucial for chemotherapy dose modifications. Management plans should be individualized according to the patient’s clinical condition.

## 1. Introduction

Myeloid sarcoma (MS), a subtype of acute myeloid leukemia (AML), is defined as a tumor mass consisting of myeloid blasts, with or without maturation, occurring at an anatomical site other than the bone marrow [1]. Dusenbery et al. reported that of the 1832 patients enrolled in subsequent Children Cancer Group (CCG) trials, 10.9% of the patients had evidence of MS at diagnosis [2]. The most commonly reported location was the skin, followed by orbits, other head and neck sites, and brain or spinal cord [2]. MS of the gastrointestinal tract is rare and is reported in only 7% of adults with MS [3]. Seven pediatric patients presenting with jaundice secondary to MS have been reported in the literature [4,5,6,7,8,9]. Here, we report the case of a patient presenting with obstructive jaundice secondary to MS of the pancreas, managed by modified doses of chemotherapy without relief of biliary obstruction.

## 2. Case Description

**Figure 1:**
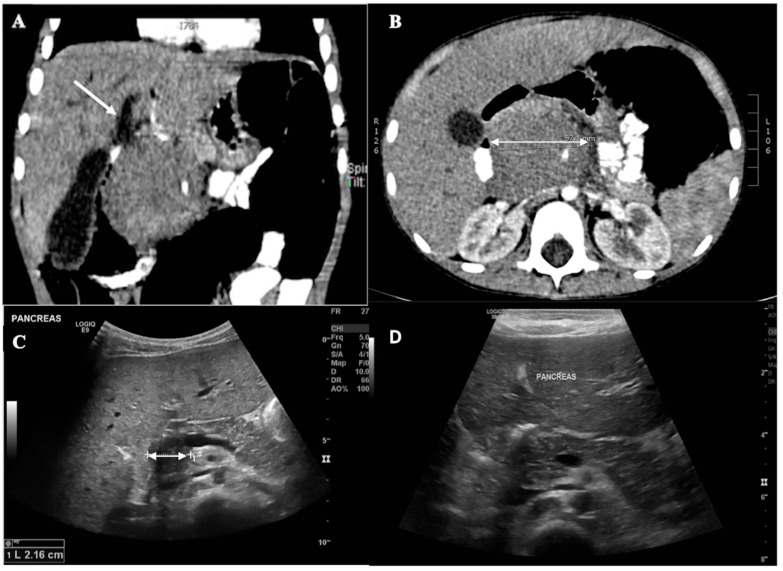
(**A**,**B**): Axial and coronal contrast enhanced CT images of the abdomen show a well-defined large round homogenous mass at the region of the pancreatic head measuring 5.2 cm × 5.7 cm × 6.1 cm displacing adjacent structures and encasing superior mesenteric artery, with upstream biliary tree dilatation (arrow). (**C**): Greyscale axial ultrasound image at the level of pancreatic head after starting chemotherapy obtained on day 17 shows mass size reduction measuring 3.4 cm × 3 cm × 2 cm, with reduced upstream biliary tree dilatation. (**D**): Grey scale ultrasound image after induction 2 showed no songoraphic residual mass, with resolved biliary tree dilatation.

A 4-year-old boy, previously healthy, presented to a peripheral hospital with a 2-week history of progressive jaundice. His initial complete blood count at the referring hospital was reported as normal with no abnormal cells seen. He was initially diagnosed clinically with Hepatitis A and treated conservatively. No virology studies were reported at the time. However, his symptoms persisted over subsequent weeks, along with the development of fever, dark urine, pale stools, and severe pruritus. Physical examination revealed no palpable masses, lymphadenopathy, or hepatosplenomegaly. An abdominal ultrasound showed a mass at the peripancreatic head region causing biliary obstruction. Abdominal computed tomography showed a well-defined retroperitoneal mass which was displacing the pancreatic head anteriorly and compressing the common bile duct with biliary dilatation (Figure 1A,B).

The patient was referred to the pediatric surgeons in our cancer center to biopsy the mass with the impression of an isolated pancreatic tumor. Upon admission, 6 weeks after his symptoms started, initial investigations showed a white blood cell count 20 × 10^9^/L, hemoglobin level 93 gm/L, platelet count 426 × 10^9^/L, and 32% blasts in the peripheral smear. His liver function tests (LFTs) revealed the following levels: total bilirubin 17.9 mg/dL (306 μmol/L), direct bilirubin 16.6 mg/dL (284 μmol/L), gamma-glutamyl transferase 209 IU/L, alanine transaminase 136 IU/L, aspartate aminotransferase 88 IU/L, and alkaline phosphatase 851 IU/L. His initial LDH was 503 mmol/L and uric acid 87 μmol/L. Virology studies showed Hepatitis A IgM antibodies were nonreactive while IgG was reactive. Bone marrow aspirate showed 46% blast infiltration, with flow cytometry results positive for MPO, CD13, CD33, and CD117 diagnostic for AML with monocytic differentiation (AML-M5) and cytogenetics positive for t(8;21) (q22;q22) and loss of chromosome Y. NPM1 and FLT1 mutation were both negative. Cerebrospinal fluid was negative for malignant cells. The conclusion was that the peripancreatic mass was an MS causing obstructive jaundice. The patient was to receive chemotherapy treatment as per the AML Medical Research Council-15 protocol [10]. The induction phase of the protocol consisted of Ara-C in addition to the hepatotoxic drugs; etoposide and daunorubicin.

We sought to relieve the common bile duct obstruction before starting chemotherapy. Endoscopic retrograde cholangiopancreatography (ERCP) was not available in the hospital, surgical intervention was associated with concerns of complications and delayed chemotherapy, and radiation therapy-associated complications outweighed the benefits. We, therefore, proceeded with systematic chemotherapy without a biopsy of the mass. The patient was started on induction therapy with ten days of full doses of Ara-C and five days of 50% dose reduction of etoposide. His bilirubin level gradually decreased after starting chemotherapy, reaching 4.6 mg/dL (79 μmol/L) by day 9 of treatment. He received 50% of the dose of daunorubicin on day nine and then full doses on days 10 and 11. He tolerated chemotherapy well, and his LFTs gradually improved. Bone marrow evaluation after the first cycle of chemotherapy showed that the patient was in remission with negative minimal residual disease. Sequential abdominal ultrasonography showed interval size regression of the mass (Figure 1C). Once the patient was due for the second induction cycle, his LFTs normalized, and he received full doses of chemotherapy. After the second induction cycle, an ultrasound showed complete resolution of the mass (Figure 1D). He completed chemotherapy smoothly with three cycles of consolidation and was in remission four years after therapy was completed.

## 3. Discussion

MS reportedly occurs in four situations: as a herald to AML in a non-leukemic patient, as a sign of impending blast crisis in chronic myeloid leukemia or leukemic transformation in myelodysplastic disorders, as an additional manifestation in patients with known AML, or as an isolated event [11]. The etiology of MS remains unclear. The current theories are focused on cell adhesion molecules, chemokine receptors/ligand interactions, and aberrant FAS-MAPK/ERK signaling [12]. The cell adhesion molecules studied include CD56 and CD11b [12]. CD56 is expressed on adipose/soft tissue, gastrointestinal tract, skeletal muscles, and the brain, which are common sites of MS. Furthermore, studies showed that CD56-positive blast cells were similar in patients with AML with and without MS [13]. CD11b is selectively expressed on mononuclear cells, and has an increased expression in AML with monoblastic/myelomonocytic differentiation, an AML subtype that is associated with increased risk of MS [13]. However, this reported finding may reflect an association rather than a causation [12]. Studies on different chemokine receptors/ligand interactions included studies on CCR5 in cutaneous tissue and the blast expression for its ligand CCL3, and the bone marrow specific chemokine CXCR4 and CXCR7 and its skin ligand CXCL12 [14]. The data on somatic loss of RAS-MAPK/ERK signaling negative regulator or metastasis-suppressor RAF kinase inhibitor protein has been reported to be non-convincing [12].

MS shows no sex, age, or site predilection, and the symptoms at presentation are diverse, depending on the size and site of the mass [15]. The diagnosis of MS causing obstructive jaundice is challenging by radiographic examination alone, difficult to biopsy, and rarely suspected in a non-leukemic patient. Our patient initially presented with obstructive jaundice secondary to a localized mass, with no reported abnormalities in the complete blood count. Once myeloid blast cells were observed in the peripheral blood weeks after the initial presentation and the bone marrow results confirmed a diagnosis of AML, we concluded that the peripancreatic mass was an MS.

There is no consensus on the treatment protocol of MS due to absence of randomized controlled trials. Isolated MS progresses to AML in 90% of untreated patients after approximately 5–12 months [16,17]. Therefore, the literature recommends treatment should be based on systematic chemotherapy to all patients: AML with MS, isolated MS, or MS after complete surgical resection [16,17]. The management of patients with AML and obstructive jaundice is challenging [18]. Daunorubicin and etoposide are primarily metabolized in the liver, with daunorubicin excreted mainly through the hepatobiliary system [19]. Protocols advise dose modification or holding of the drug in the presence of elevated bilirubin levels [10]. However, these protocols assume hepatic pathologies rather than obstructive pathologies. The alternatives to reduce bilirubin levels before chemotherapy include ERCP, surgical intervention, or localized radiation therapy. ERCP is a valid option to relieve the obstruction rapidly; however, it comes with a risk of complications, including pancreatitis, infection, bleeding, and perforation [20]. ERCP was not an option for our patient as there was no available pediatric endoscope. Surgical intervention is not without complications, particularly bleeding and infection, which may delay starting chemotherapy [21]. Local radiation therapy of MS did not appear to contribute to the overall survival rate [15]. In addition, radiation in a child may result in unnecessary morbidity, particularly if the mass is expected to be chemo-sensitive. 

Review of the literature has shown seven cases in the pediatric age group who presented with jaundice secondary to MS (Table 1). Treatment was not initiated in two patients due to their poor general condition and they succumbed to their diseases [4,5]. Another two patients received full doses of cytarabine alone as induction therapy and showed a rapid drop in bilirubin levels without biliary drainage, indicating a rapid response of the MS to chemotherapy. Both patients ended in remission [4,6]. One patient underwent percutaneous transhepatic cholangiogram with drain insertion followed by the administration of cytarabine and 50% dose reduction of daunorubicin [7]. One patient received daunorubicin, cytarabine, and 6 mercaptopurine with no clear details on dosing used. This patient had multiple relapses and died of the disease [8]. In addition, one patient received an induction course of chemotherapy as per protocol, without elaboration on dose modifications [9]. Our patient was started on full doses of cytarabine, modified doses of etoposide, and escalating doses of daunorubicin, which were all well tolerated.

The most common mutation and translocation described in MS are mutated NPM1 and t(8:21), respectively [22,23]. There is uncertainty regarding the value of t(8;21) in MS, even though it has a favorable prognostic significance for AML [24]. Inversion-16 translocation is associated with MS, particularly in abdominal sites [25]. 

The prognostic effect of MS in pediatric AML is controversial. Old studies published had variable outcomes due to inclusion of CNS leukemia with MS in the analysis [26,27]. Kim et al. and Stove et al. reported no significant difference in event free survival (EFS) and overall survival (OS) regardless of the presence or absence of MS [28,29]. A study from India reported the EFS and OS were better in patients with AML and MS compared to those with AML without MS, and the six patients with isolated MS had 100% EFS and OS [30]. Analysis of 345 patients with isolated MS in the SEER database showed that patients with MS had a better survival rate than those without MS [31]. Sambroska et al. reported worse outcome in patients with isolated MS compared to patients with MS with AML (56% and 84%, respectively) [24]. Other studies considered MS a poor prognostic factor with lower EFS and OS [32,33]. A multivariate analysis on pediatric patients with AML revealed MS was an unfavorable prognostic factor in terms of EFS (Hazard ratio 1.67) and OS (Hazard ratio 1.623) [33]. The variability in the prognosis between different studies may be due to ethnicity, different treatment protocols, and molecular and cytogenetic characteristics. Medullary relapse after therapy was the most commonly reported site [29,34].

The risk factors for relapse include older age, increasing number of tumors, and treatment with local measures alone [35,36]. Most studies concorded that MS of the orbit and CNS have better outcome compared to other sites, while MS of the skin and bone have a higher risk of relapse [30,32,34]. Dusenbery et al. reported that pediatric patients with non-skin MS had better event-free survival than patients without MS [2]. Some studies reported patients with MS occurring in the pelvis, genitourinary tract, or gastrointestinal tract did better than those with MS occurring in other sites [31,37].

## 4. Conclusions

Our case report suggests that AML-directed chemotherapy with modifications in patients with obstructive jaundice secondary to MS may be feasible without requiring invasive testing, temporary drainage procedures, or upfront radiotherapy. Daily follow-up of LFTs is crucial for chemotherapy dose modifications. The management plan should be individualized according to the patient’s clinical condition, the presence or absence of other lesions, risk factors for infection, and any associated organ dysfunction.

## Figures and Tables

**Table 1:** Pediatric cases with myeloid sarcoma and obstructive jaundice.

	Author	Age (years)/Gender	Diagnosis/Cytogenetics	Clinical Presentation	Location of Chloroma	Blasts in PBS/BM Involved	Diagnosis	Isolated vs. Disseminated	Management during Acute phase with Jaundice	Outcome
1	Our patient	4/M	Acute myeloid leukemia, M5 t(8;21)	Obstructive jaundice	Head of pancreas	Initially negative, blasts appeared after 6 weeks Yes	BMA	Isolated	Cytarabine with modified etoposide, then daunorubicin added	Remission—off therapy for 3 years
2	Rajeswari et al. [4]	1/F	Acute myeloid leukemia, likely M5 N/A	Pancytopenia that initially resolved. 2 months later presented with pancytopenia, obstructive jaundice, ascites, pale stools, fever	Soft tissue between liver and pancreas	No Not done	Ascitic tap flow cytometry	Isolated	Cytarabine	Died due to sepsis
3	Rajeswari et al. [4]	0.8/F	Acute myeloid leukemia N/A	Jaundice, dark urine, pale stool, LAP, HSM, bilateral parotid swelling	Malignant stricture, no mass visualized	Yes Not done	Peripheral blood film, MPO positive blasts, parotid gland biopsy	Isolated	None due to poor clinical condition	Died due to disease
4	Kawamura et al. [5]	15/F	Acute myeloid M2, relapse post HSCT t(8;21) FLT3-ITD	Gut GVHD followed by jaundice	Thickening of CBD wall and obstruction	No MRD positive in BM, no morphologic abnormalities	Autopsy showed multiple MS of CBD, head of pancreas, stomach	Disseminated	None	Died of sepsis
5	Jaing et al. [6]	4/M	Acute myeloid leukemia M4 N/A	Jaundice, right upper quadrant pain, pallor hepatomegaly	Head of pancreas	Yes Yes	BMA	Isolated	Cytarabine monotherapy	Remission 15 months from diagnosis post HSCT
6	Beck et al. [7]	17/M	Acute myeloid leukemia Inversion 16, CBFB-MYH11	Jaundice, right upper quadrant abdominal pain, anorexia	Soft tissue thickening causing CBD stricture	Initially negative, blasts appeared after 2 weeks Yes	BMA	Isolated	Biliary drainage by PTC followed by chemotherapy (50% dose reduction of daunorubicin, full dose cytarabine)	Remission 2 years from diagnosis
7	Mwanda et al. [8]	3.5/M	Acute myeloid leukemia N/A	Jaundice, Pallor, anorexia, vomiting, fever, abdominal distention	Pancreas	Initially negative, blasts appeared after 3 months Yes	BMA Initial biopsy from mass was not conclusive	Isolated	Daunorubicin, cytarabine, 6-mercaptopurine	Achieved remission then developed relapse in both MS and BM twice and died from disease 14 months after diagnosis
8	Miri-Aliabad et al. [9]	3/F	Acute myeloid leukemia (M2) t(8;21)	Jaundice, Fever, abdominal pain	Pancreas	No Yes	BMA	Isolated	AML MRC-12 induction protocol	Remission 1 year after completion of treatment

AML, acute myeloid leukemia; BMA, bone marrow aspirate; CBD, common bile duct; F, female; GVHD, gut versus host disease; HSCT, hematopoietic stem cell transplantation; HSM, hepatosplenomegaly; LAP, lymphadenopathy; M, male; MPO, myeloperoxidase; MRC, Medical Research Council; MRD, minimal residual disease; MS, myeloid sarcoma; N/A, not available; PTC, percutaneous transhepatic cholangiogram.

## Data Availability

Not applicable.
